# Case Report: Pediatric *Rickettsia felis* encephalitis—a rare case and literature review

**DOI:** 10.3389/fped.2026.1867339

**Published:** 2026-07-27

**Authors:** Qianqian Zhang, Meifang Lei, Hong Li, Gui Yi, Dong Li

**Affiliations:** Department of Pediatric Neurology, Children’s Hospital, Tianjin University/Tianjin Children’s Hospital, Tianjin, China

**Keywords:** doxycycline, encephalitis, infection, pediatrics, *Rickettsia felis*

## Abstract

**Background:**

*Rickettsia felis* (*R. felis*), an obligate intracellular bacterium, has been reported to cause human encephalitis. Clinical reports of *R. felis* encephalitis remain rare, particularly in children. Herein, we present a pediatric case and review the relevant literature.

**Case report:**

A previously healthy 9-year-old boy initially presented with fever and headache. Following admission, he developed hyperpyrexia and somnolence. Cranial magnetic resonance imaging revealed a left temporal lobe lesion with ipsilateral temporoparietal meningeal enhancement, and electroencephalography showed background slowing. Metagenomic next-generation sequencing of cerebrospinal fluid detected a high abundance of *R. felis* sequences, whereas autoantibody testing for central nervous system autoimmune diseases was negative. Based on these findings, a diagnosis of *R. felis* encephalitis was established. The child fully recovered and was discharged after receiving doxycycline-based antimicrobial therapy combined with glucocorticoids, intravenous immunoglobulin, and intracranial pressure management.

**Conclusion:**

This rare case highlights that *R. felis* infection should be included in the differential diagnosis of encephalitis. Metagenomic next-generation sequencing is recommended for early etiological diagnosis to facilitate timely and effective clinical intervention.

## Background

*Rickettsia felis* (*R. felis*) is a zoonotic pathogen first identified in 1990 (1). Human and animal infections have been reported worldwide (2), with cat fleas (*Ctenocephalides felis*) serving as the primary vector. Common clinical manifestations include fever, headache, malaise, and rash, whereas eschar is rare. Some patients present with fever as the sole symptom (3). In rare cases, the pathogen can invade the central nervous system (CNS) and cause encephalitis. Pediatric cases are uncommon, with only three reported to date (4–6).

## Case report

A previously healthy 9-year-old boy was admitted to our hospital on December 11, 2025, with a 3-day history of progressively worsening, predominantly frontal headache accompanied by vomiting and one episode of fever. On the day of admission, his temperature rose to 38.0 °C without other symptoms. He lived in a bungalow with a domestic dog, and numerous stray cats were noted in the surrounding area. Physical examination revealed stable vital signs and unremarkable cardiopulmonary, abdominal, and neurological findings. No rash or eschar was observed.

Cranial computed tomography (CT) on admission was unremarkable. Routine blood tests showed no significant abnormalities, whereas C-reactive protein was elevated to 21.9 mg/L (reference range, 0–8 mg/L), erythrocyte sedimentation rate to 44 mm/h (reference range, 0–15 mm/h), and serum interleukin-6 to 127.6 pg/mL (reference range, 0–7 pg/mL). Procalcitonin remained within the normal range. Liver and kidney function, myocardial enzymes, coagulation parameters, immunoglobulins, and complement levels were all normal. Lymphocyte subsets, assessed by flow cytometry, were also unremarkable. Empirical intravenous acyclovir therapy was initiated, along with mannitol for cerebral edema. Despite these measures, no clinical improvement was observed.

On day 2, video electroencephalography revealed background slowing with sharp-shaped slow waves over the bilateral frontopolar and frontal midline regions.

**Figure 1:**
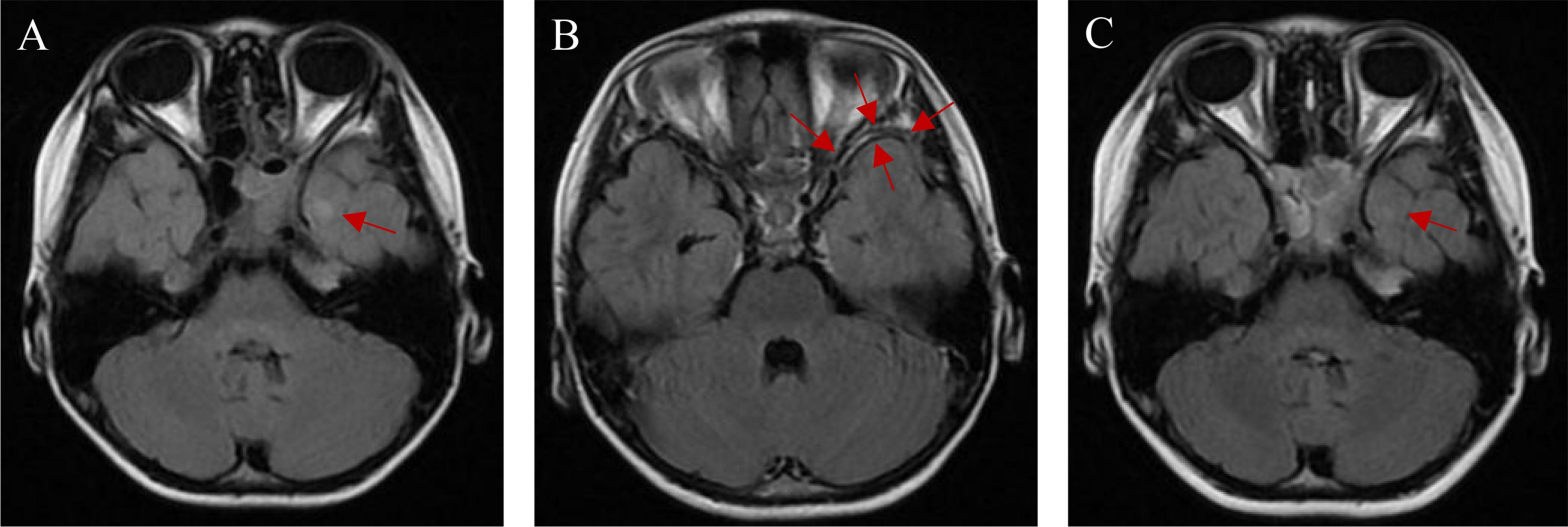
Brain MRI findings in a child with *R. felis* encephalitis. **(A)** FLAIR imaging on hospital day 3 demonstrating a hyperintense lesion in the left temporal lobe; **(B)** post-contrast image on hospital day 3 revealing meningeal enhancement in the left temporal lobe; **(C)** FLAIR imaging on hospital day 20 showing resolution of the lesion.

On day 3, the patient deteriorated with hyperpyrexia (41.3 °C), chills, and worsening headache. Brain magnetic resonance imaging (MRI) demonstrated a small hyperintense lesion in the left temporal lobe on T2-weighted and fluid-attenuated inversion recovery (FLAIR) sequences (Figure 1), whereas magnetic resonance angiography was unremarkable. Post-contrast imaging revealed meningeal enhancement in the ipsilateral temporoparietal region (Figure 1), while the temporal lesion did not enhance. Given the suspicion of viral encephalitis or CNS autoimmune disease, intravenous methylprednisolone was initiated. A repeat C-reactive protein level of 87.1 mg/L prompted the addition of intravenous cefotaxime for empirical antimicrobial coverage.

On day 4, the child developed somnolence and nuchal rigidity. At this point, a clinical diagnosis of encephalitis could be made, and lumbar puncture was recommended to identify its etiology.

On day 5, following parental consent, lumbar puncture revealed an opening pressure of 224.4 mmH_2_O and clear, colorless cerebrospinal fluid (CSF) with normal routine and biochemical findings. The frequency of mannitol administration was increased to facilitate intracranial pressure control.

On day 7, CSF metagenomic next-generation sequencing (mNGS) detected *R. felis* with 7,364 reads, a relative abundance of 4.1%, and high confidence, covering 254,368 bp of the genome at 16.1% coverage and 1.4× mean depth. In addition, a total of five microorganisms, including one pathogen and several suspected background microorganisms, were detected by mNGS; the complete list is provided in Supplementary Table S1. The raw mNGS data of CSF have been deposited in the Genome Sequence Archive for Human (GSA-Human) (29, 30), under accession number HRA019233. CSF cytokines were markedly elevated, with interleukin-6 at 1,265.51 pg/mL (reference range, 0–6 pg/mL) and interleukin-8 at 2,035.91 pg/mL (reference range, 0–52.8 pg/mL). Antibody testing for CNS autoimmune diseases was negative. Based on these findings, a diagnosis of *R. felis* encephalitis was established. Intravenous doxycycline (2 mg/kg q12 h) was initiated, and acyclovir and cefotaxime were discontinued. Temperature normalized. The Weil-Felix test was negative 3 days later.

On day 8, intravenous immunoglobulin was administered at a total dose of 2 g/kg over 3 days for immunomodulation.

On day 9, somnolence improved and nuchal rigidity resolved.

On day 11, headache resolved and serum C-reactive protein normalized.

On day 19, background activity on video electroencephalography normalized.

On day 20, brain MRI showed resolution of the left temporal lobe lesion (Figure 1). Repeat CSF analysis demonstrated normal opening pressure, no detectable *R. felis* by mNGS, and normalized interleukin-6 and interleukin-8 levels.

On day 24, having completed a 17-day course of intravenous doxycycline without any observed adverse reactions, the patient was discharged after a full clinical recovery.

## Discussion

*R. felis* is classified within the transitional group of the genus *Rickettsia* and is an obligate intracellular Gram-negative bacterium. First discovered in 1990 (1) and named in 1996 (7), *R. felis* has a global distribution, with up to 53 species serving as vectors (8), among which the cat flea is the primary vector. The pathogen infects various animals and humans (2). Since the first human case was reported in the United States in 1994 (9), cases have been documented on all continents except Antarctica (10). Exposure to dogs and cats infested with cat fleas may be a risk factor for human infection (11), and this factor was present in the pediatric patient described here.

Pathogens of the genus *Rickettsia* are introduced through inoculation by the bite of a feeding tick or mite, or by scratching infected louse or flea feces on the skin into the skin. Following inoculation, the organisms infect local CD68⁺ cells (macrophages and/or dendritic cells), migrate through lymphatic vessels to regional lymph nodes, and subsequently disseminate hematogenously to microvascular endothelial cells throughout multiple organs, including the brain, skin, lungs, liver, and other organs (12). At the cellular level, rickettsiae adhere to and invade host cells through interactions between their surface antigens and host receptors; once internalized, they exploit actin polymerization for intracellular movement and cell-to-cell spread (12). These infection processes, together with the ensuing inflammatory response, cause microvascular endothelial dysfunction and increased vascular permeability—the pathological basis for variable clinical manifestations of rickettsioses. The pathogen may enter the CNS via a transcellular route, causing encephalitis (13). However, the cellular and molecular mechanisms underlying *R. felis* pathogenesis remains to be elucidated (11).

*R. felis* can invade the CNS and cause encephalitis, a rare event. To date, 19 cases have been reported across 14 studies worldwide (4–6, 14–24), including only three pediatric cases (Table 1). Most patients present with fever, whereas only one case exhibits a rash, and none has an eschar. Clinical manifestations range from mild to severe. Neuroimaging findings suggest that lesions may involve the cerebral cortex, white matter, basal ganglia, thalamus, meninges, and small cerebral vessels. Some patients exhibit elevated CSF pressure, and routine and biochemical analyses typically reveal findings consistent with aseptic inflammation. In summary, *R. felis* encephalitis lacks characteristic clinical and ancillary findings. In the present case, rash and eschar were absent; brain MRI showed a left temporal lobe lesion and left temporoparietal meningeal enhancement. Although CSF pressure was elevated, routine and biochemical findings remained normal, consistent with previous reports. Furthermore, the patient developed hyperpyrexia, which coincided with markedly elevated CSF interleukin-6 levels. The high levels of both interleukin-6 and interleukin-8 in this patient's CSF are linked to the expression and secretion of various cytokines and chemokines induced by rickettsial infection of cerebral microvascular endothelial cells (12)—a phenomenon rarely reported.

**Table 1:** Summary of clinical features in *R. felis* encephalitis case reports.

Study	Case	Gender/Age	Symptoms	Cranial imaging involvement sites	CSF					Diagnostic basis	Antimicrobial therapy	Prognosis
					Pressure mmH_2_O	WBC ×10^6^/L	MNCs %	Protein g/L	Glucose mmol/L			
Zhang et al. (4), 2025	1	F/10	Fever headache photophobia somnolence	MRI, Bilateral frontal and parietal lobes, basal ganglia, and meninges	320	950	99	1.08	N	tNGS	Doxycycline	Recovery
Li et al. (5), 2023	1	M/8	Fever seizures impaired consciousness	MRI, Bilateral cerebral cortex and basal ganglia	—	47	56	0.49	N	mNGS	Azithromycin	Improvement
Li et al. (6), 2026	1	F/9	Fever, dizziness, irritability	MRI, Contrast-enhanced CT, N. CSF	210	36	97	0.52	3.07	mNGS	Doxycycline	Recovery
Qiu et al. (14), 2024	1	F/36	Impaired consciousness	MRI, N	170	N	—	N	N	NGS	Azithromycin	Recovery
Wang et al. (15), 2023	2	F/23	Fever headache	—	415	260	—	1.51	1.5	mNGS	Doxycycline	Improvement
		M/29	Fever headache	MRI, Unaffected	350	46	—	—	—	mNGS	Doxycycline	Recovery
Ye et al. (16), 2021	1	M/24	Fever Rash seizures impaired consciousness	CT, N	—	8	25	0.33	3.6	mNGS	Doxycycline	Improvement
Zeng et al. (17), 2021	1	F/26	Fever headache left facial and left limb paresthesia	MRI, Right thalamus	150	6	—	0.2	—	mNGS	Doxycycline	Improvement
Mawuntu et al. (18), 2020	2	M/32	Fever headache impaired consciousness	Contrast-enhanced CT, Meninges	—	122	95	2.0	1.7	Nested PCR	Anti-TB	Death
		M/38	Fever impaired consciousness	—	—	5	10	0.5	1.3	Nested PCR	Anti-TB	Death
Lindblom et al. (19), 2010	2	M/47	Fever headache photophobia phonophobia	Contrast-enhanced CT, N	—	—	—	0.41	—	Nested PCR	Not used	Recovery
		F/89	Fever and confusion	CT, N	—	—	—	—	—	Nested PCR	Cefotaxime	Improvement
Jin et al. (20), 2025	3	M/60	Vertigo	MRI, Meninges and multiple small intracranial vessels	160	46	96	1.18	2.44	NGS	Doxycycline	Improvement
		M/55	Fever	—	190	1,282	22	2.65	N	NGS	Minocycline	Improvement
		M/84	Fever abnormal behavior cognitive decline	MRI, Multiple small intracranial vessels	80	N	—	N	N	NGS	Doxycycline	Improvement
Zhang et al. (21), 2021	1	F/18	Fever seizures impaired consciousness	MRI, Bilateral frontal, parietal, temporal, and insular lob	224.4	N	—	N	N	mNGS	Minocycline, Levofloxacin	Improvement
Luo et al. (22), 2025	1	F/25	Fever headache	MRI, N	320	1	—	0.29	3.3	mNGS	Doxycycline	Recovery
Wang et al. (23), 2025	1	M/36	Headache mental abnormalities	MRI, Meninges	—	375	88	0.78	—	mNGS	Doxycycline	Recovery
Wang et al. (24), 2023	1	M/33	Fever seizures cognitive decline	MRI, Bilateral amygdala and hippocampus	160	162	93	0.7	N	mNGS	Doxycycline	Improvement
This study^a^	1	M/9	Fever headache somnolence	MRI, Left temporal lobe, meninges	224.4	2	50	0.23	3.48	mNGS	Doxycycline	Recovery

MNCs, mononuclear cells; anti-TB, anti-tuberculosis; —, indicates “Data not available”; N, indicates “normal range”.

a Present case.

The diagnosis of *R. felis* infection relies on serological and molecular methods. Serological methods include the Weil–Felix test, indirect immunofluorescence assay, and enzyme-linked immunosorbent assay. The Weil–Felix test is no longer recommended due to its poor sensitivity and specificity; in this case, it yielded a false-negative result. In addition, the latter two methods, because of cross-reactivity, limit species-specific identification and require paired acute- and convalescent-phase sera for antibody titer comparison (25), thereby impeding early diagnosis. Molecular techniques include polymerase chain reaction (PCR) and next-generation sequencing (NGS). PCR is limited to detecting predefined target sequences and has relatively low sensitivity. By contrast, pathogen NGS encompasses mNGS and targeted NGS (tNGS). mNGS involves direct extraction of total nucleic acids (human cells and microorganisms) from the sample, followed by unbiased library preparation, sequencing, and bioinformatics analysis (including quality control, host read removal, and microbial alignment), thereby enabling broad-spectrum pathogen detection. A key advantage of mNGS is its ability to identify rare, novel, or uncultivable pathogens, making it especially valuable when conventional methods fail to detect the causative agent, particularly in critically ill or diagnostically challenging patients (26). Because *R. felis* is a rare, fastidious, and difficult-to-culture pathogen and its associated encephalitis lacks characteristic features, mNGS enables effective diagnosis of this condition, although low-abundance organisms may escape detection. tNGS targets dozens to hundreds of known pathogen genomes, enriching target sequences via PCR amplification or probe hybridization, thus offering higher sensitivity and specificity but reduced flexibility (26). In this case, the diagnosis was confirmed by detecting *R. felis* in CSF using mNGS. Notably, all reported cases of *R. felis* encephalitis since 2021 have been diagnosed using NGS of CSF, further highlighting its clinical utility.

Doxycycline remains the drug of choice for *R. felis* infection in all patients, including pregnant women and children. Given its high oral bioavailability, the oral route is generally preferred. However, intravenous administration may be warranted in patients with severe nausea and vomiting or in those who are critically ill and unable to tolerate oral therapy. The recommended treatment duration is 7 days (27). In this case, the duration of intravenous therapy may have been longer than necessary; transition to oral doxycycline could have been considered once the patient's condition had stabilized. The American Academy of Pediatrics has stated that short-term doxycycline (≤21 days) can be used regardless of age, and a clinical study has demonstrated that such use is safe in children under 8 years (28). No adverse reactions were observed in this case. Overall, most patients with *R. felis* encephalitis have a favorable prognosis, with few fatalities. The patient described here achieved complete clinical recovery and was discharged.

## Conclusion

*R. felis* encephalitis rarely presents with rash or eschar, and neuroimaging and routine CSF findings are often nonspecific, making diagnosis challenging. Therefore, clinicians should enhance their awareness of this infection and maintain a high index of suspicion. Early diagnosis by mNGS enables timely intervention and may reduce the risk of severe neurological complications and mortality.

## Data Availability

The raw sequence data reported in this paper have been deposited in the Genome Sequence Archive (Genomics, Proteomics & Bioinformatics 2025) in National Genomics Data Center (Nucleic Acids Res 2025), China National Center for Bioinformation / Beijing Institute of Genomics, Chinese Academy of Sciences (GSA-Human: HRA019233) that are publicly accessible at https://ngdc.cncb.ac.cn/search/specific?db=hra&;q=PRJCA067058. Accession: PRJCA067058

## References

[1] Adams JR Schmidtmann ET Azad AF. Infection of colonized cat fleas, Ctenocephalides felis (Bouché), with a rickettsia-like microorganism. Am J Trop Med Hyg. (1990) 43(4):400–9. 10.4269/ajtmh.1990.43.400 2240368

[2] Tsokana CN Kapna I Valiakos G. Current data on Rickettsia felis occurrence in vectors, human and animal hosts in Europe: a scoping review. Microorganisms. (2022) 10(12):2491. 10.3390/microorganisms10122491 36557744 PMC9781214

[3] Angelakis E Mediannikov O Parola P Raoult D. Rickettsia felis: the complex journey of an emergent human pathogen. Trends Parasitol. (2016) 32(7):554–64. 10.1016/j.pt.2016.04.009 27155905

[4] Zhang M Zhu X Lu X Jiang L Chen J Diao Y. Rickettsia felis meningoencephalitis in a 10-year-old child: a case report and literature review. BMC Pediatr. (2025) 25(1):901. 10.1186/s12887-025-06230-2 41188777 PMC12584534

[5] Li WJ Zhao Y Cao BB Zheng KL Shi KL Li Y. Rickettsia felis encephalitis in children: a case report and literature review. Chin J Clin Neurosci. (2023) 31(2):204–8, in Chinese.

[6] Li X, Jiang Y, Dai R, Yang Y, Wang W. Rickettsia felis meningoencephalitis in a child: a case report and literature review. Front Pediatr. (2026) 14:1763281. 10.3389/fped.2026.1763281 42038247 PMC13106386

[7] Higgins JA Radulovic S Schriefer ME Azad AF. Rickettsia felis: a new species of pathogenic rickettsia isolated from cat fleas. J Clin Microbiol. (1996) 34(3):671–4. 10.1128/jcm.34.3.671-674.1996 8904435 PMC228867

[8] Zhang YY Sun YQ Chen JJ Ai-Ying T Tao W Simon IH. Mapping the global distribution of spotted fever group rickettsiae: a systematic review with modelling analysis. Lancet Digit Health. (2023) 5(1):e5–15. 10.1016/S2589-7500(22)00212-6 36424337 PMC10039616

[9] Schriefer ME Sacci JB Jr Dumler JS Bullen MG Azad AF. Identification of a novel rickettsial infection in a patient diagnosed with murine typhus. J Clin Microbiol. (1994) 32(4):949–54. 10.1128/jcm.32.4.949-954.1994 8027348 PMC267160

[10] Legendre KP Macaluso KR. Rickettsia felis: a review of transmission mechanisms of an emerging pathogen. Trop Med Infect Dis. (2017) 2(4):64. 10.3390/tropicalmed2040064 30270921 PMC6082062

[11] Minahan NT Wu WJ Tsai KH. Rickettsia felis is an emerging human pathogen associated with cat fleas: a review of findings in Taiwan. J Microbiol Immunol Infect. (2023) 56(1):10–9. 10.1016/j.jmii.2022.12.006 36585292

[12] Sahni A Fang R Sahni SK Walker DH. Pathogenesis of rickettsial diseases: pathogenic and immune mechanisms of an endotheliotropic infection. Annu Rev Pathol. (2019) 14:127–52. 10.1146/annurev-pathmechdis-012418-012800 30148688 PMC6505701

[13] Sekeyová Z Danchenko M Filipčík P Fournier PE. Rickettsial infections of the central nervous system. PLoS Negl Trop Dis. (2019) 13(8):e0007469. 10.1371/journal.pntd.0007469 31465452 PMC6715168

[14] Qiu J Feng H Liu L Zhu J. A case of Rickettsia felis infection-induced encephalitis in a pregnant woman. World J Emerg Med. (2024) 15(2):150–2. 10.5847/wjem.j.1920-8642.2024.019 38476524 PMC10925534

[15] Wang J Zhou H Dong Z Wang J Wang R Guan Y. Diagnosis of two meningitis cases caused by Rickettsia felis in China, with metagenomic next-generation sequencing: a case report. Infect Drug Resist. (2023) 16:7239–45. 10.2147/IDR.S417787 38023405 PMC10656866

[16] Ye G Yang L Xu L Pan Z Dong Z. Neurological presentations caused by Rickettsia felis infection. Br J Hosp Med. (2021) 82(6):1–2. 10.12968/hmed.2021.0212 34191568

[17] Zeng Z Wang C Liu C Meng X Chen Y Guo S. Follow-up of a Rickettsia felis encephalitis: some new insights into clinical and imaging features. Int J Infect Dis. (2021) 104:300–2. 10.1016/j.ijid.2020.12.090 33444751

[18] Mawuntu AHP Johar E Anggraeni R Feliana F Bernadus JBB Safari D. Rickettsia felis identified in two fatal cases of acute meningoencephalitis. PLoS Negl Trop Dis. (2020) 14(2):e0007893. 10.1371/journal.pntd.0007893 32069292 PMC7048312

[19] Lindblom A Severinson K Nilsson K. Rickettsia felis infection in Sweden: report of two cases with subacute meningitis and review of the literature. Scand J Infect Dis. (2010) 42(11–12):906–9. 10.3109/00365548.2010.508466 20735330

[20] Jin J Dong F Xu E Wu D. Central infection with Rickettsia felis complicated with stroke: a case series and review. IDCases. (2025) 41:e02341. 10.1016/j.idcr.2025.e02341 40837615 PMC12362360

[21] Zhang L Zhang J Hu M Feng X. Severe encephalitis caused by infection of Rickettsia felis: a case report. Zhonghua Wei Zhong Bing Ji Jiu Yi Xue. (2021) 33(4):491–3. 10.3760/cma.j.cn121430-20201110-00709 34053497

[22] Luo Z Yan LL Yan R Yang YF Zhou XJ Zheng YS. A case of Rickettsia felis infection-associated encephalitis. Chin J Infect Dis. (2025) 43(4):242–4, in Chinese. 10.3760/cma.j.cn311365-20240802-00230

[23] Wang SS Li X Tang XD Ding XF Xie XB Zou GY. A case of mental disorder caused by feline rickettsial encephalitis was diagnosed by metagenomic next-generation sequencing. Chin J Lab Med. (2025) 48(6):754–7, in Chinese. 10.3760/cma.j.cn114452-20240904-00490

[24] Wang N Weng SW Shao XQ Li ZM Wang Q Lyu RJ. Neurosyphilis complicated with Rickettsia felis infection:one case report and literature review. Chin J Contemp Neurol Neurosurg. (2023) 23(4):368–74, in Chinese. 10.3969/j.issn.1672-6731.2023.04.016

[25] Blanton LS. The rickettsioses: a practical update. Infect Dis Clin North Am. (2019) 33(1):213–29. 10.1016/j.idc.2018.10.010 30712763 PMC6364315

[26] Zhao Y Zhang W Zhang X. Application of metagenomic next-generation sequencing in the diagnosis of infectious diseases. Front Cell Infect Microbiol. (2024) 14:1458316. 10.3389/fcimb.2024.1458316 39619659 PMC11604630

[27] Caravedo Martinez MA Ramírez-Hernández A Blanton LS. Manifestations and management of flea-borne rickettsioses. Res Rep Trop Med. (2021) 12:1–14. 10.2147/RRTM.S274724 33574726 PMC7873028

[28] Stultz JS Eiland LS. Doxycycline and tooth discoloration in children: changing of recommendations based on evidence of safety. Ann Pharmacother. (2019) 53(11):1162–6. 10.1177/1060028019863796 31280586

[29] Zhang S Chen X Jin E Wang A Chen T Zhang X. The GSA family in 2025: a broadened sharing platform for multi-omics and multimodal data. Genom Proteom Bioinform. (2025) 23(4):qzaf072. 10.1093/gpbjnl/qzaf072 PMC12451262 40857552

[30] CNCB–NGDC Members and Partners. Database resources of the national genomics data center, China national center for bioinformation in 2026. Nucleic Acids Res. (2026) 54(D1):D28–47. 10.1093/nar/gkaf1172 41359036 PMC12807729

