# Supplementary material for: Case Report: Pediatric Rickettsia felis encephalitis—a rare case and literature review
Source: Front Pediatr. 2026 Jul 27;14:1867339. doi: 10.3389/fped.2026.1867339 (PMC13454761; doi:10.3389/fped.2026.1867339)
Supplement: Supplementary file 1 [file Table1.docx]

Supplementary Table 1. Microorganisms detected by mNGS in cerebrospinal fluid

|  | scientific name | reads | relative abundance | confidence | genome sequence | | | classification |
| --- | --- | --- | --- | --- | --- | --- | --- | --- |
|  |  |  |  |  | total length | coverage | mean depth |  |
| Special pathogens (Mycoplasma, Chlamydia, Rickettsia, Spirochetes, etc.) | *Rickettsia felis* | 7364 | 4.1% | high | 254,368 bp | 16.1% | 1.4× | Pathogen |
| Bacteria | *Acinetobacter junii* | 12,006 | 6.7% | - | 460,446 bp | 13.4% | 1.2× | Suspected background microorganism (colonizer) |
|  | *Comamonas testosteroni* | 1681 | 1.0% | - | 45,812 bp | 0.8% | 1.0× | Suspected background microorganism (environmental organism) |
|  | *Rothia mucilaginosa* | 158 | 0.1% | - | 6526 bp | 0.3% | 1.1× | Suspected background microorganism |
| Fungi | *Trichosporon mucoides* | 115 | 0.8% | - | 5675 bp | 0.01% | 1.0× | Suspected background microorganism |
| Mycobacteria | Not detected |  |  |  |  |  |  |  |
| Viruses | Not detected |  |  |  |  |  |  |  |
| Parasites | Not detected |  |  |  |  |  |  |  |
| Note: –, data not provided. | | | | | | | | |
